# Deep mendelian randomization: Investigating the causal knowledge of genomic deep learning models

**DOI:** 10.1371/journal.pcbi.1009880

**Published:** 2022-10-20

**Authors:** Stephen Malina, Daniel Cizin, David A. Knowles

**Affiliations:** 1 Department of Computer Science, Columbia University, New York, New York, United States of America; 2 Dyno Therapeutics, Watertown, Massachusetts, United States of America; 3 Tri-Institutional Ph.D. Program in Computational Biology and Medicine, Weill Cornell Medicine, New York, New York, United States of America; 4 New York Genome Center, New York, New York, United States of America; 5 Department of Systems Biology, Columbia University, New York, New York, United States of America; 6 Data Science Institute, Columbia University, New York, New York, United States of America; Massachusetts General Hospital, UNITED STATES

## Abstract

Multi-task deep learning (DL) models can accurately predict diverse genomic marks from sequence, but whether these models learn the causal relationships between genomic marks is unknown. Here, we describe Deep Mendelian Randomization (DeepMR), a method for estimating causal relationships between genomic marks learned by genomic DL models. By combining Mendelian randomization with *in silico* mutagenesis, DeepMR obtains local (locus specific) and global estimates of (an assumed) linear causal relationship between marks. In a simulation designed to test recovery of pairwise causal relations between transcription factors (TFs), DeepMR gives accurate and unbiased estimates of the ‘true’ global causal effect, but its coverage decays in the presence of sequence-dependent confounding. We then apply DeepMR to examine the global relationships learned by a state-of-the-art DL model, BPNet, between TFs involved in reprogramming. DeepMR’s causal effect estimates validate previously hypothesized relationships between TFs and suggest new relationships for future investigation.

## Author summary

Chromatin marks such as transcription factor (TF) binding, accessibility, and histone modifications play a critical role in controlling cell behavior and identity. In recent years, multi-task deep learning (DL) models have achieved remarkable success at predicting these and other chromatin marks. However, it is unclear to what extent these models learn meaningful mechanistic, even causal, relationships between these variables. Our work aims to fill this gap by combining *in silico* mutagenesis, deep learning uncertainty estimation and causal inference (specifically Mendelian randomization, MR), into a framework we call DeepMR. We describe DeepMR, apply it to a simulation intended to test its ability to recover causal relationships between features from a learned model, and then use it to examine the relationships learned by a state-of-the-art DL model, BPNet. Our results suggest that DeepMR can estimate causal relationships under its stated assumptions and provide further evidence for previously hypothesized relationships between TFs identified by BPNet.

## Introduction

Deep learning (DL) has achieved success predicting genomic marks such as transcription factor (TF) binding [1, 2], chromatin accessibility [2, 3], histone modifications [4], RNA binding protein (RBP) binding [1, 5–7] and splicing [8, 9] from DNA (or RNA) sequence. These models, often convolutional neural networks [10], typically achieve high predictive accuracy and recognize sequence features that match those found by orthogonal experiments such as SELEX [11]. In particular, multi-task models such as DeepSEA [2] and BPNet [12] can accurately predict multiple genomic marks simultaneously. Following [13], we define a “mark” as a position in the genome where the number of reads from an epigenomic assay is significantly above background. Here we ask: do such multi-task models, through learning to predict multiple marks jointly, gain an implicit understanding of mechanistic, causal relationships between marks?

We attempt to answer this question by developing Deep Mendelian Randomization (DeepMR). DeepMR combines *in silico* mutagenesis with Mendelian randomization (MR) [14], an instrumental variable approach for causal inference, to estimate learned causal effects in genomic DL models. DeepMR obtains local (sequence level) and global (genome level) estimates of (an assumed) linear causal relationship between pairs of marks learned by a multi-task genomic prediction model. DeepMR draws on four threads of work spanning machine learning and statistical genetics.

### DL for functional genomics

A major objective in functional genomics is mapping sequence-to-function relationships between genotype and molecular phenotypes, typically leveraging large-scale observational data from high-throughput assays such as ChIP-seq [15–18], DNase-seq [19], and ATAC-seq [20]. Understanding this mapping enables 1) better understanding of epigenomic regulation, 2) variant interpretation, and 3) more accurate prediction of downstream traits. However, achieving these goals requires decoding complex relationships between high-dimensional genomic sequence inputs and interrelated outputs from large, noisy datasets. Encouraged by DL models’ ability to overcome similar challenges in the fields of computer vision and natural language processing, genomics researchers have trained DL models on functional genomics datasets with substantial success.

Early work showed that DL could predict sequence-to-function relationships accurately and demonstrated their promise for identifying trait-associated variants. DeepBind [1], one of the earliest DL sequence-to-function classifiers, outperformed then state-of-the-art models at predicting TF binding and RBP binding from sequence. DeepBind and other classification models—e.g. DeepSEA [2] and Basset [3]—also identified trait-associated variants with higher accuracy than previous methods. More recent work has leveraged DL models to improve our understanding of epigenomic regulatory logic. In particular, [12] trained a regression model, BPNet, to predict the binding of four TFs and used it to dissect the motif-based regulatory grammar that governs their binding. Together, these papers illustrate the promise of DL models for not only predicting function from sequence but also improving our understanding of epigenomic regulation and ability to anticipate disease risk. In our work, we seek evidence that genomic DL models learn meaningful high-level relationships between output marks.

### Model interpretation

Local interpretation methods characterize how specific input (sequence) features influence predictions or intermediate layer activations (e.g., saliency maps [21], guided back-propagation [22], DeepLIFT [23], and DeepSHAP [24]). Even DeepLIFT, which was designed with genomic DL in mind, focuses on interpreting individual model predictions for a single output rather than discovering relationships between outputs and is therefore complementary to our work.

Closer to our work, [25]’s Global Importance Analysis (GIA) assesses the global effect size of different patterns on model predictions. While resembling DeepMR in terms of its focus on global effects, GIA allows users to test narrower hypotheses about specific features such as motifs and uses synthetic instead of observed sequences. As such, GIA is also complementary to DeepMR, potentially providing a method for uncovering specific patterns that explain higher level relationships discovered by DeepMR.

Saturation *in silico* mutagenesis characterizes how a model’s predictions for a specific input change as a result of all possible point mutations to the input. Saturation mutagenesis has been used to assess the learned representations of genomic DL models such as DeepBind [1], cDeepBind [6], DeepSEA [2], and Basset [3]. Here, we use saturation mutagenesis (with uncertainty estimates generated using Deep Ensembles [26]) to generate a set of estimated variant *effect sizes* which we then provide as input to MR.

### Uncertainty estimates and coverage of DL predictions

Many methods for obtaining uncertainty estimates from DL models exist [27]. Our work is not focused on testing different uncertainty estimation methods so we chose Deep Ensembles [26], which, despite their simplicity, consistently perform well in empirical comparisons [26, 28]. Briefly, a Deep Ensemble is a collection of DL models trained from different random initializations, which leads to different learned weights, resulting in slightly different predictions for each data point. Deep Ensembles provide uncertainty estimates in the form of variance between the different submodels’ predictions for a given data point. Despite this, [29] found that uncertainty estimates from Deep Ensembles were often miscalibrated but could be rescued using isotonic regression (a solution we adopt here).

### Mendelian randomization

MR is an instrumental variable [30] technique for estimating (typically linear) causal effects in the presence of potential unobserved confounders. Instrumental variable approaches enable causal inference in the presence of unobserved confounding by taking advantage of *instruments*, auxiliary variables associated with the purported cause but independent of any confounding. MR was originally developed for inferring causal effects from population-scale observational data (i.e., genome-wide association studies, GWAS). MR takes advantage of genetic variation inducing population-level phenotypic variation that is independent of post-natal confounders to infer unbiased causal effects. In an early demonstration of MR, [14] used MR to estimate the effect of C-Reactive Protein on insulin resistance. In contrast to prior studies, their results suggest that C-Reactive Protein levels may not have a meaningful effect on insulin resistance. While previous studies sought to control for confounding directly, [14] use a Single Nucleotide Polymorphism (SNP) in the CRP gene as the genetic variant (instrument) for their MR analysis, which they have higher confidence lacks any association with post-natal confounders. This is one of several examples in which MR helped infer a more accurate estimate of an underlying causal effect.

Here we explore MR’s application to estimating causal effects implied by model-generated “data” with *in silico* mutations taking the place of true genetic variants. Putative causes and effects are genomic marks such as TF binding or chromatin accessibility. Applied to variant effects predicted by accurate machine learning models, MR allows us to infer the strength of relationships between phenotypes despite these relationships being confounded by the influence of other, potentially unobserved marks. While MR is traditionally applied to observed rather than estimated effects, our work attempts to show that effects estimated by DL models can satisfy the assumptions (described below) required for valid MR estimates.

**Fig 1:**
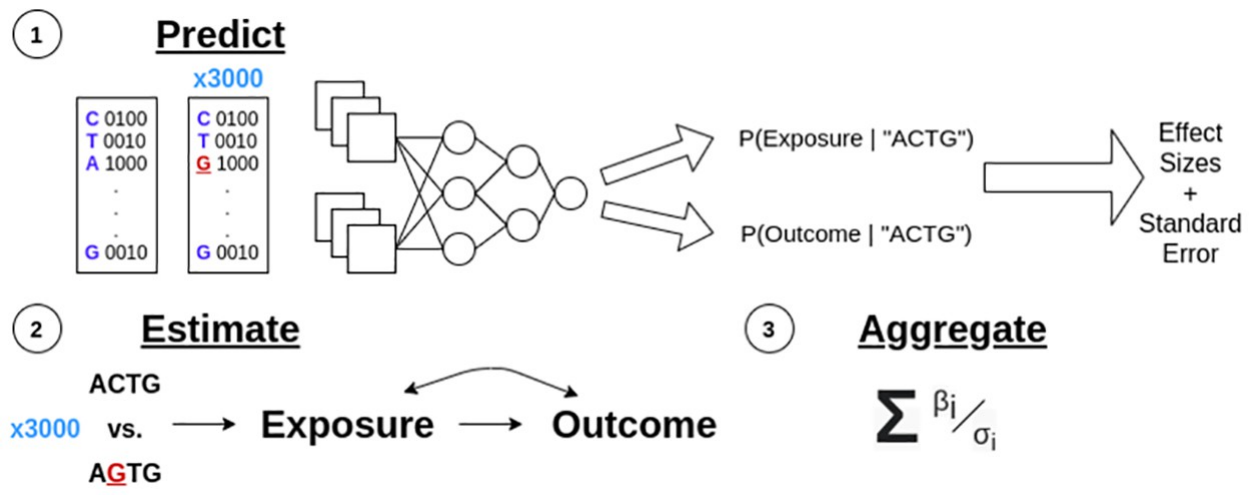
Graphical representation of DeepMR’s high-level steps. DeepMR combines *in silico* mutagenesis and Mendelian randomization (see Algorithm overview). Predict corresponds to steps 1 through 4. Estimate corresponds to step 5. Aggregate corresponds to step 6.

MR only produces valid causal effect estimates under specific assumptions (Fig 1 under Estimate) [14]. Let *Z* be a variable we intend to use as an instrument (a genetic variant for example), *X* a purported cause (*exposure*), and *Y* a purported effect (*outcome*), and suppose that there may be unobserved confounding between *X* and *Y*, denoted by *U*. Then, MR gives an unbiased estimate of the causal effect of *X* on *Y* if:

1. *Z* is independent of *U* (**Unconfoundedness**),
2. *Z* is not independent of *X*, and
3. *Z* only influences *Y* through *X* (**Exclusion Restriction**).

Early MR studies assumed that all MR assumptions were perfectly satisfied and therefore that a single instrument was sufficient for inferring a causal effect. In this setting, exposure-to-outcome causal effects can be inferred via either the Wald ratio [31] or two-stage least squares regression [30, 32]. The Wald ratio is computed as the instrument-to-outcome regression coefficient divided by the instrument-to-exposure coefficient. In two-stage least squares, we still perform an instrument-to-exposure regression but then regress the outcome onto the predicted rather than observed exposure values. The resulting coefficient is the causal effect estimate. With a single instrument, two-stage least squares produces identical results to the Wald ratio but has the advantage of being compatible with multiple instruments.

Recently developed MR methods such as Robust Adjusted Profile Score [33], MR-Egger [34], and the modal-based estimator [35] leverage multiple instruments to relax some of these assumptions without compromising the validity of results.

DeepMR can work with any MR method that takes multiple instruments’ effect sizes and standard errors as inputs and can produce effect size estimates and confidence intervals for those estimates. In this work, we estimate causal effects using 1) a simple baseline where we take the average of the Wald ratios for each instrument and 2) a robust variant of MR-Egger with the goal of being robust to invalid instruments. MR-Egger seeks robustness to violations of Exclusion Restriction, otherwise known as horizontal pleiotropy in statistical genetics [36]. MR-Egger is based on an analogy between MR with multiple instruments and meta-analysis. It treats each instrument as a ‘study’ enabling violations of exclusion restriction (Assumption 3) to be viewed as a form of small study bias. As long as the strength of the instrument-exposure relationship is independent of the direct effect of the instrument on the outcome, MR-Egger gives accurate estimates of causal effects in the presence of instruments that violate exclusion restriction.

## Methods

### Algorithm overview

DeepMR estimates causal effects between variables predicted by a multi-task model. It takes a trained, calibrated (regression or classification) model that outputs predictive means and standard errors and a set of one-hot encoded sequences as input. It outputs local, sequence-specific causal effects and global, exposure/outcome-specific causal effects. It accomplishes this (see Fig 1 for a visual depiction) via the following steps for each exposure/outcome pair:

1. Randomly sample sequences to predict exposure and outcome values for “reference sequences”.
2. Perform saturation *in-silico* mutagenesis for each reference sequence to generate (sequence length × alphabet size − 1) mutated sequences per reference sequence.
3. For each set of pairs of mutant and reference sequences, generate predictive means and standard errors for exposure and outcome features.
4. Generate (sequence length × alphabet size − 1) *effect sizes* by subtracting each reference sequence’s predictive mean from the corresponding mutated sequences’ predictive means. Additionally, compute the standard errors of these differences.
5. Filter instruments by effect size based on a *z*-score threshold (Assumption 2) to only include those that are strongly associated with the exposure.
6. Estimate a per-sequence region causal effect by running MR on the remaining effect sizes and their standard errors.
7. Estimate global causal effects using a random effects meta-analysis across sequence regions (loci).

#### Exposure and outcome effect size & standard error estimation

Step 4 requires variant effect estimates for each mutation for both the exposure *X* and outcome *Y*. Let *f*_*X*_(*Z*, *θ*^(*i*)^) and *f*_*Y*_(*Z*, *θ*^(*i*)^) be the model for *X* and *Y* respectively with input sequence *Z* and parameters *θ*^(*i*)^ representing the *i*th component of the deep ensemble. Appealing to the interpretation of a deep ensemble as an approximation to a posterior predictive distribution [37], the posterior expectation for *X* is $E\left[X|Z\right]\approx \frac{1}{N}\sum_{i=1}^{N}f_{X}(Z,\theta^{\left(i\right)})$. Calculating this Monte Carlo (MC) estimate for both the mutant sequence *m* and reference *r* we can obtain an unbiased estimate of the variant effect ${\hat{\beta }}_{ZX}=E(X|Z=m)-E\left[X|Z=r\right]$. We proceed analogously for the outcome *Y*.

A naive estimate of the standard errors (s.e.) would use $\text{var}[{\hat{\beta }}_{ZX}]=\text{var}\left[X|Z=m\right]+\text{var}\left[X|Z=r\right]$ with the variances estimated by MC. However, this would give inflated s.e. since it ignores statistical dependence resulting from *θ*. We therefore instead use

$$\begin{array}{cc} \text{var}[{\hat{\beta }}_{ZX}]=\; & \text{var}[(X|Z=m)-(X|Z=r)]\\ = & \frac{1}{N}\text{var}\left[X|Z=m\right]+\text{var}[X|Z=r]-2\text{cov}[X|Z=m,X|Z=r]\\ = & \frac{1}{N}\sum_{i=1}^{N}{\left[f_{X}(m,\theta^{\left(i\right)})-E[X|Z=m]\right]}^{2}+\\ & \frac{1}{N}\sum_{i=1}^{N}{\left[f_{X}(r,\theta^{\left(i\right)})-E[X|Z=r]\right]}^{2}-\\ & 2(\frac{1}{N}\sum_{i=1}^{N}[f_{X}(m,\theta^{\left(i\right)})f_{X}(r,\theta^{\left(i\right)})]-E[X|Z=m]E[X|Z=r]). \end{array}$$

We again proceed analogously for the outcome *Y*.

#### Per sequence region causal effect estimation

For the per-sequence region causal effect estimation, we treat the subset of ${\hat{\beta }}_{ZX},{\hat{\beta }}_{ZY}$ pairs and their accompanying s.e. values that passed the step 5 filter (i.e. the mutation is associated with *X*) as input to the chosen MR method. From this, MR provides us with one causal effect estimate and associated s.e. per sequence region.

To estimate a global causal effect, we apply a random effects meta-analysis to the per-sequence region causal effects and their s.e. values. Briefly, a random effects meta-analysis assumes that the true effect for each study (sequence region) is drawn from an underlying global distribution of effect sizes, which is typically assumed to be Gaussian whose mean and variance are to be estimated. Per-sequence region effect are observed with mean equal to the the true effect and Gaussian noise with variance determined by the s.e. from MR. To perform the random effects meta-analysis, we use the meta R package [38].

### Simulation

Our simulation is inspired by [39] but tailored to test DeepMR’s ability to estimate the strength of the causal relationship between exposure and outcome TFs when binding to simulated *L* = 100bp DNA sequences. The exposure TF’s binding affinity, *c*_*e*_, is determined primarily by the probability of the TF (represented as a position weight matrix, PWM) binding anywhere on the sequence (see S1 Text), *p*_*e*_,

$$\begin{array}{c} c_{e}=\alpha p_{e}+\eta p_{c}+\tau z+1, \end{array}$$ (1)

where *p*_*c*_ is the binding probability of an optional confounder TF, and *z* ∼ Bernoulli(0.5) is an optional sequence independent confounder. By contrast, the outcome TF’s binding affinity *c*_*o*_ is a multiplicative function of both the strength of its own motif match and the strength of the exposure’s, i.e.

$$\begin{array}{c} c_{o}=\alpha \gamma p_{o}p_{e}+\nu p_{c}+\tau z+1. \end{array}$$

Here the effect size *γ* represents the influence of the exposure’s binding on the outcome’s binding in raw counts space. *γ* is not the *true causal effect* because the true CE is defined in Anscombe-transformed rather than raw counts space. *γ* is sampled (once per simulation run) from an equal proportion mixture of two normals with means 10 and 1 (and variance 0.5), in order to test DeepMRś ability to differentiate between two clusters of CEs, one much lower than the other. We sample *α* from *N*(100, 3) (once per simulation run) and fix *η* = 20, *ν* = 30 and *τ* = 25.

The simulation model corresponds to a causal effect of the exposure TF on the outcome TF: with no exposure TF binding there can be no outcome TF binding. When present, both types of confounding influence exposure and outcome counts multiplicatively.

Finally to represent experimental noise, counts are Poisson distributed with mean equal to the affinity values. We did not use a negative binomial since we expect the random sequence generation process will naturally induce overdispersion. Finally, we Anscombe transform the raw counts [39].

Length 100 sequences are sampled uniformly at random. For each TF, with 50% probability we insert a subsequence sampled from its PWM at a random position. To assign a binding probability we convolve the TF’s PWM over the sequence and apply the soft-or function (see S1 Text).

We considered four different scenarios: 1) no unobserved confounding, 2) sequence-based unobserved confounding, 3) non-sequence-based unobserved confounding, and 4) both types of confounding in tandem. Sequence-dependent unobserved confounding adds an additional TF (and corresponding) motif which influences the binding strength of both exposure and outcome TFs.

We train ensembles of convolutional neural network (CNN) models on the data produced in each scenario and use them, combined with held-out test sets, as inputs for DeepMR.

#### True causal effect computation

To assess the quality of our method, we need to compare its estimates to the ground truth. DeepMR estimates the effect of a unit change in the exposure on the outcome by using single point mutations that meaningfully affect the exposure as instruments. Our simulation can provide us with the true affinity for any given mutated sequence, which we leverage to compute true sequence-region level causal effects. For a given sequence which contains the exposure motif, the true causal effect is found by regressing the effect of all point mutations to bases within the exposure motif on the outcome on the corresponding effects on the exposure. This is similar to the two-stage least squares MR method [32] where all mutations within the the exposure motif are assumed to be valid instruments.

#### Simulation & model parameters

In all simulation runs, we used PWMs representing motifs for the GATA (exposure), TAL1 (outcome), and SOX2 (confounder) transcription factors, all drawn from ENCODE’s motif database [40] and sampled using the simdna library (https://github.com/kundajelab/simdna).

To model this data, we trained 3-layer CNN with 15 filters per convolutional layer and a filter width of 7 for maximum 100 epochs with early-stopping. The three convolutional layers were followed by 2 hidden layers of width 30. Models were trained using Adam with a learning rate of 10^−3^ and otherwise standard parameters combined with an MSE loss to predict the Anscombe-transformed counts for the exposure and outcome jointly.

Code for all experiments can be found at https://github.com/an1lam/deepmr.

## Results

We first assess DeepMR on simulated data where we know the ground-truth relationship between the modeled TFs. We then apply DeepMR to determining the causal relationships between four TFs involved in pluripotency.

### Simulation

#### DeepMR accurately estimates global CEs in all cases

We evaluated DeepMR’s local and global CE estimates in the one unconfounded and three confounded scenarios (see Methods). In each scenario, we performed causal effect estimation (including learning the sequence-to-binding CNN ensemble) for 50 simulations using 10000 training sequences and 1000 test sequences for CE estimation. In each scenario, we compare results obtained using MR-Egger as the MR method to those obtained using a simple MR baseline of taking the average and standard deviations of the Wald ratios to produce each local CE and interval estimate respectively.

**Fig 2:**
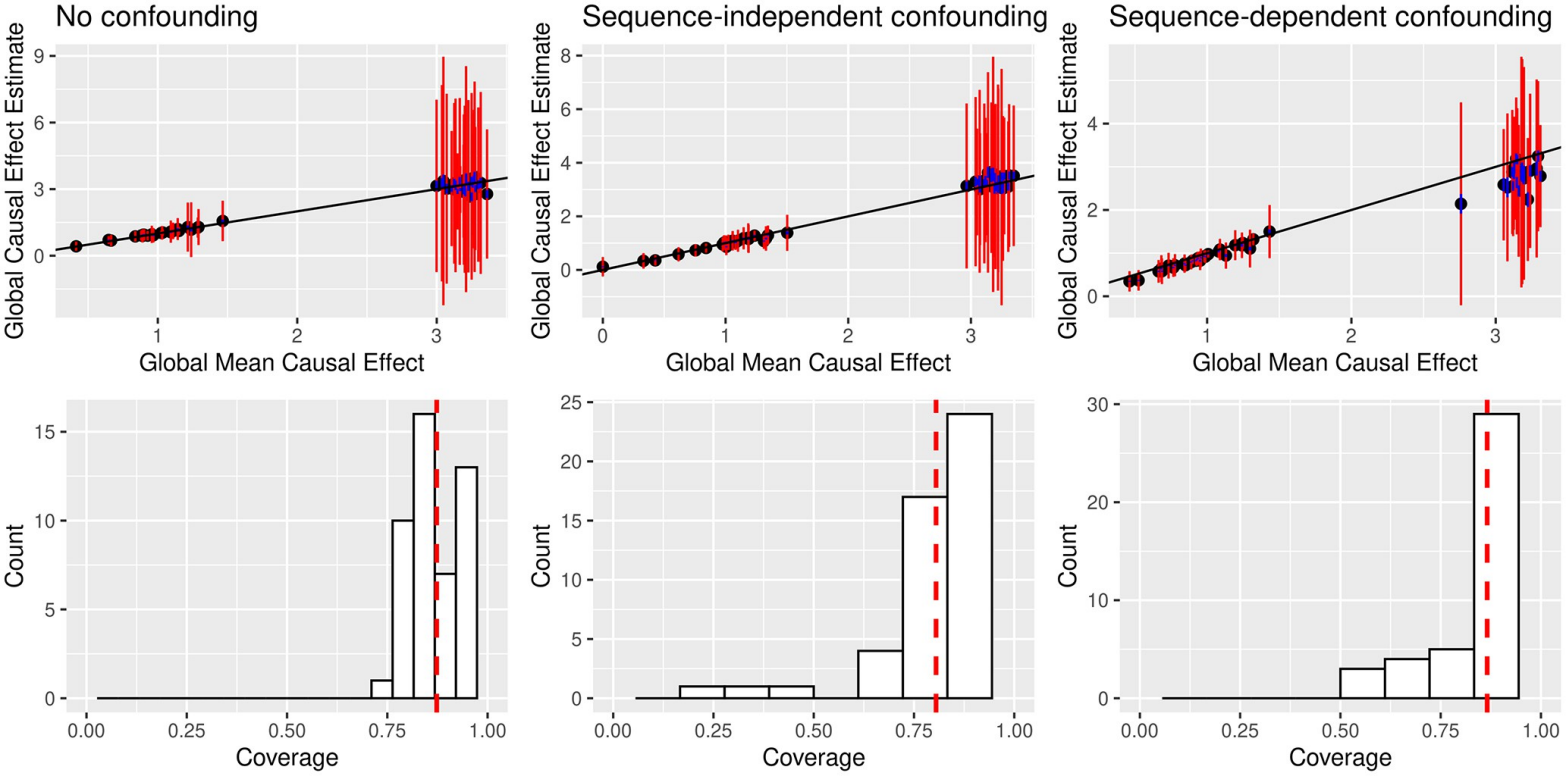
In simulations, DeepMR estimates causal effects between TFs even in the presence of unobserved confounding. **Top row**: true vs. estimated global causal effects (CEs) across 50 rounds for unconfounded, random confounded, and sequence-dependent confounded cases respectively. Blue bars denote ±2*σ* where *σ* denotes the standard error of the mean and orange bars denote ±2*τ* where *τ* denotes the between-region standard deviation. **Bottom row**: local CE coverage (how often the true CE is in the 95% confidence interval) across the three experiments (same order) with the red line denoting average coverage.

Our CNN models achieved *R*^2^ validation accuracy averaging around 0.8 for (transformed) exposure counts and 0.7 for (transformed) outcome counts. For the causal inference we assessed two metrics: accuracy of global CEs and coverage of local CE 95% confidence intervals. We judged accuracy of global CEs in terms of the correlation between the global causal effect estimates and the average of the true global causal effects and the frequency at which the CE estimate ±2*τ* capture said average across 50 simulations. DeepMR accurately estimates true global CEs in all cases (Fig 2, Table 1 for *R*^2^ accuracy values). In the unconfounded and non-sequence confounding cases, we see near-perfect agreement between estimated and true global CEs. In the sequence-dependent confounding case, DeepMR more often underestimates true CEs, although usually by less than one s.e., suggesting that the influence of the unlabeled SOX motif score on the exposure and outcome label values biases DeepMR’s global CE estimates towards 0.

**Table 1:** DeepMR estimates causal effects (CE) accurately with high coverage. Accuracy is *R*^2^. Local corresponds to CEs for individual regions, global for the meta-analysis mean. For global CE accuracy and coverage the first value comes from using MR-Egger and the second from the baseline MR procedure.

	Global CE Accuracy	Global CE Coverage	Local CE CI Coverage
Unconfounded	0.97/0.98	1.00/1.00	0.87
Random	0.98/0.97	1.00/0.96	0.81
Sequence	0.97/0.98	0.98/0.86	0.87
Both	0.78/0.96	0.98/0.92	0.81

#### DeepMR’s coverage decays in the presence of sequence-dependent confounding

We judged coverage of global CEs by measuring the fraction of ±2*τ* intervals that capture the true global CE. We judged coverage of local CEs by examining the distribution of 95% confidence interval coverage across 50 simulations in the four scenarios. DeepMR performs better in the unconfounded and random confounding scenarios. While average coverage (see Table 1) is relatively constant across scenarios, in Fig 2 we observe a longer tail of low coverage values in the random (see Table A and Table B in S2 Text for the impact of confounder strength on these metrics) and sequence-dependent confounding scenarios. Furthermore, global CE coverage in the sequence-dependent and scenario is much lower. Together, these results suggest that DeepMR can somewhat underestimate variance in confounded scenarios and produces more calibrated local CE estimates in cases where there is minimal or no sequence-dependent confounding.

#### DeepMR produces accurate estimates using both MR-Egger and the baseline

Overall, DeepMR’s estimates are accurate using both MR-Egger and the baseline estimation method. In fact, the baseline method generates more accurate estimates in the presence of both confounding types, likely because MR-Egger underestimates the CEs in this setting. However, there is seemingly a trade-off where MR-Egger provides better coverage in the presence of sequence-dependent confounding, whereas the baseline’s coverage is substantially reduced in this setting.

### Estimating causal effects between four TFs involved in reprogramming

Given the promising results on simulated data, we applied DeepMR to detecting CEs between four TFs involved in induced pluripotent stem cell (iPSC) reprogramming: Oct4, Sox2, Nanog, and Klf4. We used the ChIP-nexus data and model (BPNet) previously described in [12] but trained a 5-component ensemble. We closely followed the data processing and model training process used in the original paper, described in full at the BPNet repository (https://github.com/kundajelab/bpnet). We calibrated the resulting Deep Ensemble with isotonic regression using validation data. We computed local CE estimates for all TF pairs on 2000 randomly sampled sequences in the validation set. These estimates were used to compute global estimates for each TF pair via meta-analysis.

#### DeepMR validates previously hypothesized and suggests new relationships between TFs

**Fig 3:**
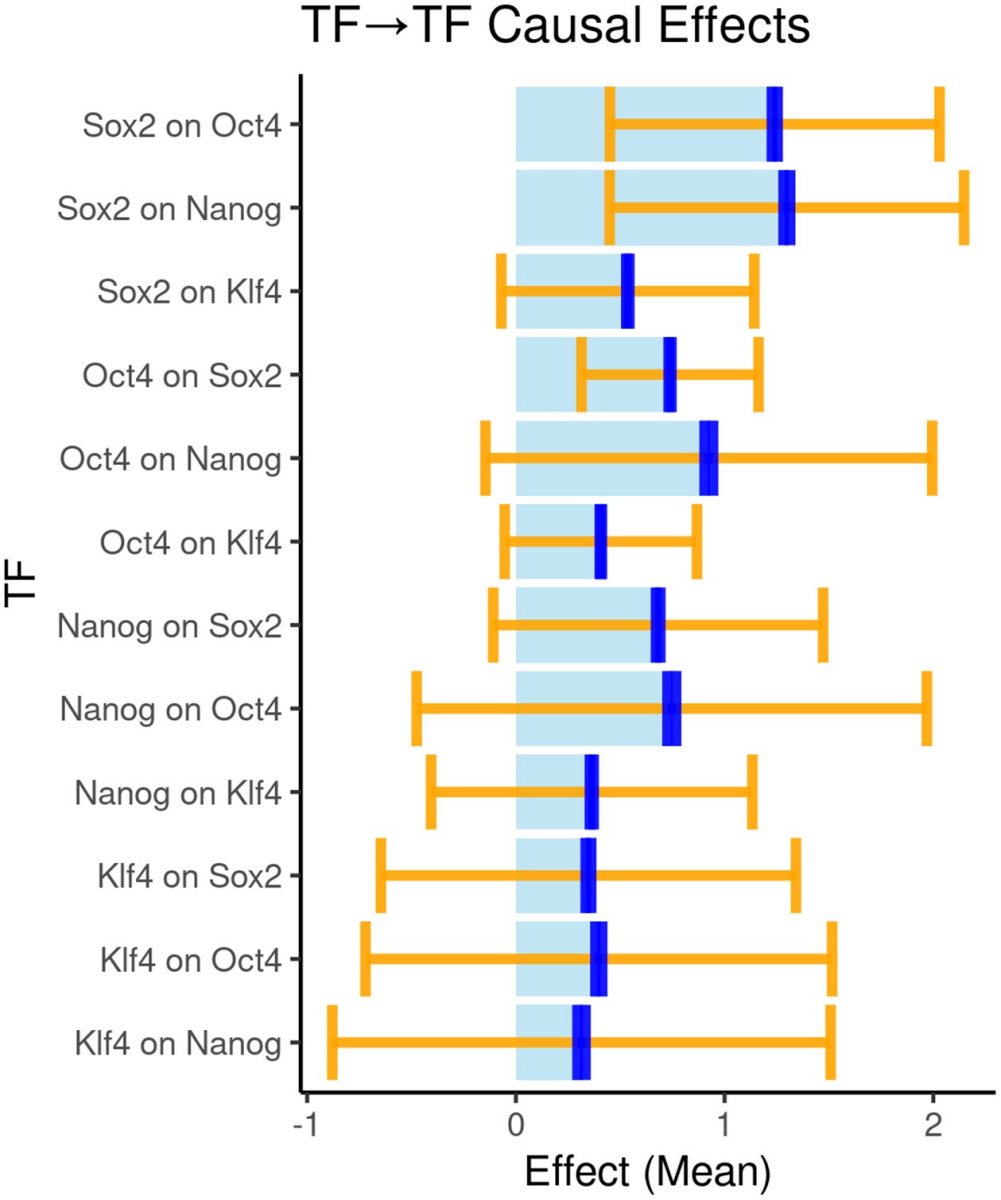
Global CE estimates. Global CEs for all pairs of TFs predicted by BPNet with ±2*τ* (orange) and ±2*σ* (blue) ranges around the mean estimate.

Based on an orthogonal approach (TF cooperativity analysis), [12] postulate a positive directional effect of a composite Oct4-Sox2 binding motif on the binding of Nanog and Klf4. As a test of DeepMR’s ability to discover such relationships while making fewer assumptions about their functional form, we sought to replicate this finding. While the BPNet approach does not produce quantitative overall estimates of directional effects, it enabled them to make two hypotheses about directionality (see [12]’s Extended Data Fig 6). These were 1) Sox2 and Oct4 act on Nanog and 2) that Oct4 and Sox2 act on each other via a composite motif. To replicate these findings, we computed the global CEs for all 12 pairs of TFs. We largely recapitulate [12] (Fig 3), finding that Sox2 and Oct4 both have a strong positive estimated CEs on each other’s binding and on the binding of Nanog. In the latter case, the 2*τ* range does include 0, suggesting high variability across loci. In general, we observe high variability across sequence regions, reflected by the generally large ±2*τ* ranges. This also matches [12]’s observation that effects vary across sequence space and in particular with different motif spacings (see S1 Fig for a heatmap showing the effect of motif spacing on global CE estimates).

DeepMR suggests additional hypotheses that could be validated by in future experimental work. As one example, DeepMR predicts that Sox2 acts on Klf4 more strongly than the reverse.

## Discussion

DeepMR estimates the magnitude of causal relationships between outputs of multi-task genomic DL models in order to hypothesize specific models of gene regulation. DeepMR can recover CEs in the presence of unobserved confounding in simulation and validates purported and identifies new putative relationships between four important TFs involved in reprogramming. While DeepMR shows promise, it does have several known limitations.

### Resource requirements

Since DeepMR relies on *in silico* mutagenesis across each submodel in the Deep Ensemble, generating the data for estimating global CEs is computationally intensive, taking approximately one day to run for the BPNet hold-out set in our experiments. One could incorporate speed-ups such as those of [41] or leverage attribution tools such as saliency maps, DeepLIFT [23] or DeepSHAP [24] that can efficiently approximate *in silico* mutagenesis.

### Model calibration

MR Egger requires properly calibrated effect size and standard error estimates for each instrument. Our ensemble-based approach to uncertainty estimation tends to produce somewhat over-confident estimates as measured by the metrics proposed by [29]. We apply and recommend isotonic regression [42] to remedy this.

### Violation of MR assumptions

For MR to return unbiased causal effect estimates, the underlying data-generating process and our model’s proxy for it must both adhere to the three MR assumptions and there must be an at least approximately linear relationship between exposure and outcome. In the statistical genetics setting, these assumptions can be justified in part by claims about the relationship between genotype, which is determined pre-natally, and potential confounders and phenotypes, which tend to manifest post-natally, assuming population structure is accounted for. We cannot fall back on these justifications for sequence-to-function relationships. Instead, we must re-examine each of these assumptions to determine whether they can be expected to hold. Assumption 2 is easily satisfied because by filtering instruments based on their relationship to the exposure (see Algorithm overview), whereas the unconfoundedness (Assumption 1), exclusion restriction (Assumption 3), and linearity assumptions have the potential to be violated.

Under classical MR assumptions, estimates will only be unbiased if all instruments are independent of unobserved confounders. Potential unobserved confounders fall into two categories: sequence-dependent and sequence-independent. Classical MR (i.e. inverse-variance weighting) should control for sequence-independent confounding. Potential sequence-dependent confounders include other TFs, chromatin features or an uncorrected assay bias such as GC-bias. Such confounders additionally violate the exclusion restriction assumption by providing a causal pathway from instrument (mutation) to outcome not mediated by the exposure TF. However, our use of MR Egger provides some additional robustness to such violations so long as the InSIDE assumption holds. Indeed, our simulation experiments (**Simulation**) showed remarkable robustness to the effects of both types of confounding.

MR correctly estimates causal effects when all relationships —instrument to exposure and exposure to outcome—are linear, which may not be the case. For example, given strong TF binding cooperativity, knocking out one TF’s binding will knock out the other’s entirely, violating linearity. Fortunately, we only require the weaker condition of local linearity. Each of our effect sizes is derived from a single mutation, so DeepMR behaves correctly so long as the relationships stay linear within a local neighborhood. Going beyond the assumption of local linearity is something we hope to address in future work.

In summary, DeepMR relies on specific assumptions about model quality and the true causal relationships. The former can be expected to increase as genomic datasets grow. The latter suggests relaxing some of these assumptions via more advanced MR methods or developing tools to detect when assumptions are violated.

In the future, we will aim to combine DeepMR with a causal network inference method such as our recent bimmer model [43] to explicitly account for the influence of other assayed TFs on each pair. DeepMR would also benefit from accompanying tools for diagnosing when model-generated data deviates from or violates MR assumptions.

## Supporting information

S1 Text Computing binding probabilities. (PDF)

S2 Text Effect of confounder strength on simulation metrics. (PDF)

S1 Fig Effect of motif spacing on BPNet global CEs. Heatmap of Global CEs broken down by TF pairs and motif spacing buckets. Each row represents the global effects of one TF on another, computed using a subset of sequences in which both TF’s motifs appeared within the relevant distance range of each other. The figure shows the effect of motif spacing on global CE estimates for the four BPNet TFs. To compute effects for each TF and spacing bin, we used motif instance annotations from [12] to select sequence regions with motif instance pairs. For each sequence region, we computed distances between the two motif instances. Finally, we binned the sequence regions by motif instance distance and ran DeepMR on the sequence regions within each bin for each pair of TFs to obtain global CEs. The horizontal color banding in the heatmap illustrates that, while motif spacing has some effect on global CEs, the inter-spacing differences tend to be much smaller than the inter-TF differences.
(TIF)
