# Supplementary material for: Deep mendelian randomization: Investigating the causal knowledge of genomic deep learning models
Source: PLoS Comput Biol. 2022 Oct 20;18(10):e1009880. doi: 10.1371/journal.pcbi.1009880 (PMC9624391; doi:10.1371/journal.pcbi.1009880)
Supplement: S1 Text — (PDF) [file pcbi.1009880.s001.pdf]

S1 Text. Computing Binding Probabilities.

Given a PWM and a DNA sequence, represented by its one-hot encoding $\mathbf{s} \in \{0,1\}^{4\times L}$, we want to obtain the probability of observing binding at any of the sequence's M-length subregions under the assumption that binding probabilities are independent across the subregions. We first compute the probability of binding at each subregion follow the process described by [1], a modified version of the energy-based model of binding proposed in [2].

To aggregate probabilities across positions into a single sequence binding probability, we let $p_{i:i+M}$ denote the probability of the TF binding at sequence subregion spanning nucleotide i to $i+M$ and compute the probability of binding somewhere on the sequence as

$$1 - \prod_{i=1}^{L-M} (1 - p_{i:i+M}).$$

That is, the probability of the TF binding somewhere is one minus the probability of it not binding anywhere. This operation is sometimes known as "soft-or".

References

1. Finkelstein M, Shrikumar A, Kundaje A. Look at the Loss: Towards Robust Detection of False Positive Feature Interactions Learned by Neural Networks on Genomic Data. In: Proceedings of the 37th International Conference on Machine Learning (ICML 2020). The 2020 ICML Workshop on Computational Biology; 2020.

2. Zhao Y, Ruan S, Pandey M, Stormo GD. Improved models for transcription factor binding site identification using nonindependent interactions. Genetics. 2012;191(3):781–790.
