# Supplementary material for: Deep mendelian randomization: Investigating the causal knowledge of genomic deep learning models
Source: PLoS Comput Biol. 2022 Oct 20;18(10):e1009880. doi: 10.1371/journal.pcbi.1009880 (PMC9624391; doi:10.1371/journal.pcbi.1009880)
Supplement: S2 Text — (PDF) [file pcbi.1009880.s002.pdf]

S2 Text. Effect of Confounder Strength on Simulation Metrics.

Confounder Strength	Global CE Accuracy	Global CE Coverage	Local CE CI Coverage
10	1.00	1.00	0.88
50	0.99	1.00	0.79
100	0.95	1.00	0.43
150	0.91	1.00	0.39

Table A. Table showing CE metrics for different random confounder coefficient values applied to the simulation. Global CE accuracy only slightly decays as the strength of the confounder increases, but local CE CI coverage decays significantly, suggesting that individual CIs fail to account for the additional noise from random confounding even if pooled estimates remain accurate.

Confounder Strength	Global CE Accuracy	Global CE Coverage	Local CE CI Coverage
20	0.97	1.00	0.93
50	0.21	0.80	0.58
100	0.88	1.00	0.32
150	0.99	1.00	0.43

Table B. Table showing CE metrics for different sequence-dependent confounder coefficient values applied to the simulation. Global CE accuracy decays as the strength of the confounder increases, but local CE CI coverage decays significantly, suggesting that individual CIs fail to account for the additional noise from random confounding even if pooled estimates remain accurate.
