# Supplementary figures and images for: Deep mendelian randomization: Investigating the causal knowledge of genomic deep learning models
Source: PLoS Comput Biol. 2022 Oct 20;18(10):e1009880. doi: 10.1371/journal.pcbi.1009880 (PMC9624391; doi:10.1371/journal.pcbi.1009880)

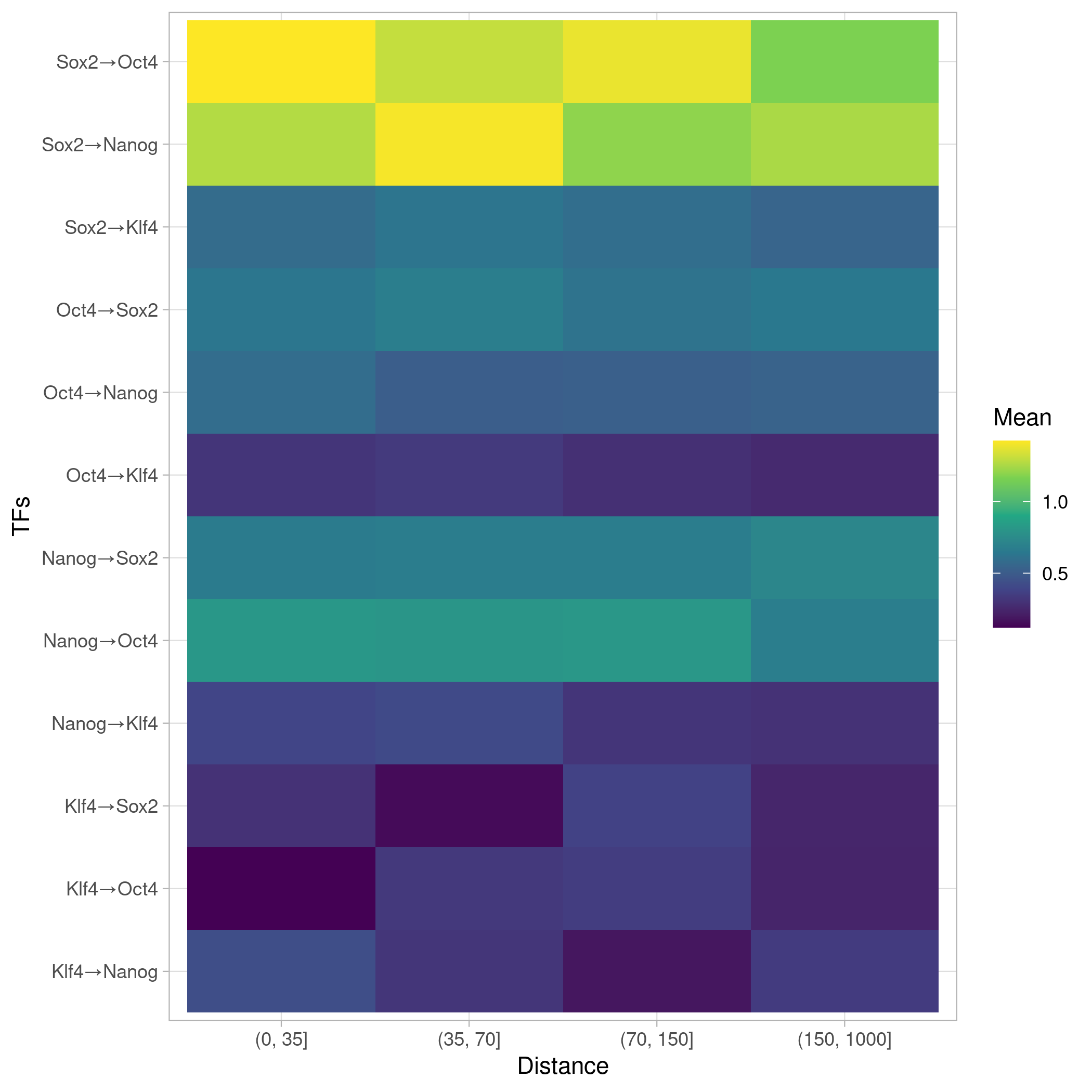

Supplement: S1 Fig — Heatmap of Global CEs broken down by TF pairs and motif spacing buckets. Each row represents the global effects of one TF on another, computed using a subset of sequences in which both TF’s motifs appeared within the relevant distance range of each other. The figure shows the effect of motif spacing on global CE estimates for the four BPNet TFs. To compute effects for each TF and spacing bin, we used motif instance annotations from [12] to select sequence regions with motif instance pairs. For each sequence region, we computed distances between the two motif instances. Finally, we binned the sequence regions by motif instance distance and ran DeepMR on the sequence regions within each bin for each pair of TFs to obtain global CEs. The horizontal color banding in the heatmap illustrates that, while motif spacing has some effect on global CEs, the inter-spacing differences tend to be much smaller than the inter-TF differences. (TIF) [file pcbi.1009880.s003.tif]
